# Use of ethanol extract of *Mycobacterium bovis* for detection of specific antibodies in sera of farmed red deer (*Cervus elaphus*) with bovine tuberculosis

**DOI:** 10.1186/1746-6148-9-256

**Published:** 2013-12-17

**Authors:** Ashutosh Wadhwa, Rachel E Johnson, Colin G Mackintosh, J Frank T Griffin, W Ray Waters, John P Bannantine, Shigetoshi Eda

**Affiliations:** 1 Department of Forestry, Wildlife and Fisheries, Center for Wildlife Health, University of Tennessee Institute of Agriculture, Knoxville, Tennessee 37996, USA; 2 AgResearch Invermay, P.O. Box 50034, Mosgiel, New Zealand; 3 Disease Research Laboratory, University of Otago, Dunedin, New Zealand; 4 Bacterial Diseases of Livestock Research Unit, National Animal Disease Center, USDA Agricultural Research Service, Ames, Iowa 50010, USA

**Keywords:** Bovine tuberculosis, ELISA, *Mycobacterium bovis*, Red deer

## Abstract

**Background:**

Bovine tuberculosis (bTB) in wildlife species poses a threat to domestic livestock in many situations. Control programs for bTB in livestock depend on testing and slaughtering the positive animals; however, the currently available diagnostic tests often have poor specificity. In our previous study, we developed a specific and sensitive enzyme linked immunosorbent assay (ELISA) for another mycobacterial disease – Johne’s disease, using surface antigens of *Mycobacterium avium* ssp. *paratuberculosis* (MAP) extracted by briefly agitating the bacilli in 80% ethanol solution. The ELISA test was named ethanol vortex ELISA (EVELISA). The objective of this study is to examine whether EVELISA technique could be used to specifically detect anti-*Mycobacterium bovis* (*M. bovis*) antibodies in the serum of *M. bovis*-infected farmed red deer (*Cervus elaphus*). We tested a total of 45 red deer serum samples, divided in 3 groups – uninfected animals (n = 15), experimentally infected with *M. bovis* (n = 15) and experimentally infected with MAP (n = 15).

**Results:**

The presence of anti-*M. bovis* antibodies was tested using an ethanol extract of *M. bovis*. Without absorption of anti-MAP cross reactive antibodies, it was found that 13 out of the 15 MAP-infected animals showed high antibody binding. Using heat killed MAP as an absorbent of cross reactive antibodies, anti-*M. bovis* antibodies were detected in 86.7% of *M. bovis*-infected animals with minor false positive results caused by MAP infection.

**Conclusions:**

The results from this study suggest that EVELISA may form a basis for a sensitive and specific test for the diagnosis of bTB in farmed red deer.

## Background

Farming of red deer (*Cervus elaphus*) has been an emerging alternative livestock industry mainly in New Zealand, USA, China, Russia and Canada [1]. Being in continuous contact with the livestock and the free-ranging wildlife, farmed red deer populations are at increased risk to get and spread infectious diseases. Bovine tuberculosis (bTB), caused by *Mycobacterium bovis* (*M. bovis*), is a chronic infectious disease of international zoonotic and economic importance [2]. It is characterized by the formation of granulomatous lesions with varying degrees of necrosis, calcification and encapsulation [3-7]. bTB has been identified in a wide range of wildlife species, domestic animals and humans [8,9]. Global economic loss due to bTB is estimated to be about US$ 3 billion annually [10]. Since there are no effective treatments or vaccines for bTB, rigorous testing and removal of diseased animals remains the only control measure.

In contrast to the control programs for bTB in wildlife species, bTB in farmed deer is primarily monitored by skin testing and rarely by slaughter surveillance. One of the major ante mortem tests for bTB is the tuberculin skin test (TST) using purified protein derivatives (PPD) of *M. bovis*[4,11]. In the US, there is requirement of a negative skin test for interstate transport and it includes a voluntary herd accreditation program [12]. However, the participation in such programs is very low due to inadequate handling facilities and need to recapture animals for testing 72 hours after the injection of PPD [13,14]. Further, a recent study showed that interpretation of TST could be confounded by infection of red deer with environmental mycobacteria [15]. An interferon-γ release assay has also been evaluated for bTB diagnosis in capture cervids, [16,17] but the test requires fresh blood samples and also has not been validated for diagnosis of bTB in free-ranging wildlife species [18]. Antibody-based assays for detection of bTB have shown promising results due to their flexibility and cost effectiveness. Prior studies on the development of antibody based assays have used cross-reactive preparations of *M. bovis*, such as a crude cell sonicate [19] culture filtrate [20] PPD [21] and lipoarbinomannan (LAM) [22]. Specific molecules like ESAT-6, CFP10, MPB83 and MPB70 have also been used for detection of anti-*M. bovis* antibodies [23-25]. Recent studies have demonstrated the advantages of using multiple antigens (e.g. ESAT-6, CFP10 and MPB83) in multi-antigen print immune-assay (MAPIA) [12,26], lateral flow rapid test (RT) [18] or dual path platform (DPP) [27] assays. Although these studies have shown promising results in detecting antibodies against *M. bovis*, the presence of anti-*Mycobacterium avium* ssp. *paratuberculosis* (MAP) antibodies due to confounding factors like infection and/or vaccination may cause interference in interpretation [28].

We have previously developed a novel enzyme-linked immunosorbent assay (ELISA), called an ethanol vortex ELISA (EVELISA) using surface antigens of MAP for detecting anti-MAP antibodies in serum at early stages of Johne’s disease (JD) [29-32]. The aim of the present work was to assess the performance of EVELISA optimized to diagnose bTB using serum samples from various groups of red deer (*Cervus elaphus*) including animals experimentally infected with *M. bovis* or MAP.

## Methods

### Samples

In order to evaluate the performance of EVELISA, a total of 45 red deer sera were obtained from 3 different studies in New Zealand. The first group (Uninfected) consisted of 15 deer approximately 12 months old, which were not challenged with either of *M. bovis* or MAP. All the animals in this group were culture negative for *M. bovis* using lymph node samples and blood samples taken one week prior to slaughter were all serologically negative for MAP using an IgG_1_ ELISA test (Paralisa™, Disease Research Laboratory (DRL), Department of Immunology and Microbiology, University of Otago, Dunedin, NZ) [33]. The second group (*M. bovis* infected) consisted of 15 deer approximately 12 months old, which had been experimentally challenged using 0.2 mL volume of 500 CFU of *M. bovis* into the left tonsillar crypt of anesthetized deer [34]. *M. bovis* was isolated at slaughter from gross lesions or pooled lymph node samples (head, thoracic or intestinal lymph nodes) from all 15 deer 27 weeks after experimental challenge. All blood samples were tested using an ELISA Tb test called EBT [35] and a comparative cervical tuberculin test (CCT) [36]. For the CCT, intradermal injections of 0.1 mL of avian tuberculin (2500 IU; A) and bovine tuberculin (5000 IU; B) were given at two closely clipped sites on the neck. Skin thickness was measured before injection and 72 hours later. The CCT is considered positive if the increase at site B is greater than or equal to site A; and, negative if site A is greater than site B. All the animals in the second group (*M. bovis* infected) were tested positive by CCT. Serum samples of 11 out of the 15 animals in this group were positive by ETB. Of the 15 blood samples collected a week prior to slaughter, 7 samples were seropositive for MAP using the Paralisa™ test. Finally, the third group (MAP infected) consisted of 15 deer experimentally infected with MAP as previously described [33]. All the samples in this group were from animals sourced from a property with no history of bTB or JD. MAP was isolated from all the deer in this group by culture after 50 weeks post infection and 12 out of the 15 samples collected immediately before slaughter were seropositive using the Paralisa™ test. Serum samples of 10 out of the 15 animals in this group were positive by ETB, showing high false positive rate in MAP infected animals. Animal use described in this study was approved by the AgResearch Invermay Ethics Committee (AEC11115).

### EVELISA

A virulent strain of *M. bovis* (HC2005T), which was originally isolated from an *M. bovis* infected dairy cow, was cultured in Middlebrook’s 7H9 medium (Becton Dickinson, Cockeysville, MD) with addition of 0.05% Tween 80 (Fisher Scientific, Fair Lawn, NJ), 10% oleic acid-albumin-dextrose-NaCl (Becton Dickinson, Microbiology Systems, Franklin Lakes, NJ) at 37°C. For antigen preparation *M. bovis* bacilli were harvested from stationary phase cultures, and centrifuged at 2,600 × *g* for 10 minutes; the pellet was then suspended in 80% ethanol and agitated by vortex at room temperature for 2 min, and centrifuged at 10,621 × *g* for 10 minutes to dislodge surface antigens. Extracted *M. bovis* antigen was diluted in the ethanol solution and 50 μL of the solution was immobilized on wells of a 96-well plate by evaporation. MAP (K10 strain) was used for preabsorption step in this study. The antigen-coated plate was incubated with 150 μL of buffer B (10 mM phosphate buffered saline, pH 7.0, containing 0.05 v/v% Tween 20 and 10 v/v% SuperBlock [PIERCE Biotechnology, Rockford, IL]) at room temperature for 30 min. The plate was then washed 4 times with 200 μL of PBST (10 mM phosphate buffered saline, pH 7.0, containing 0.05% Tween 20). Fifty μL of serum sample (with or without preabsorption of cross-reactive antibodies with heat-killed MAP [K10 strain, 4 mg/mL] for 30 minutes) was then inoculated and incubated at room temperature for one hour [32]. After washing the wells four times with 200 μL of PBST, 50 μL of horseradish peroxidase (HRP)-conjugated rabbit anti-deer IgG heavy and light chains (1:1000 dilution; Kirkegaard & Perry Laboratories, Inc. Gaithersburg, MD, diluted in buffer B) was added in each well and incubated at room temperature for one hour. After washing the wells four times with 200 μL of PBST, 100 μL of tetramethylbenzidine (TMB) solution (Thermo Scientific, Rockford, IL) was used to develop color reaction according to manufacturer’s instruction and optical density (OD) of the solution was determined by a microplate reader (Model 680, BioRad, Hercules, CA) at 450 nm for 10 min after terminating the reaction by adding 100 μL of 2 M sulfuric acid.

### Statistical analysis

All experiments were conducted in duplicate or triplicate, and repeated at least twice. The test sensitivity was determined by dividing the number of *M. bovis* culture- test positive animals by the total number of *M. bovis* culture positive animals, with the result expressed as a percentage. The test specificity was determined by dividing the number of bTB free, test-negative animals by the total number of bTB free animals, with the result expressed as a percentage. The cut-off value was determined based on the two-graph receiver operating characteristic analysis [37].

## Results

**Figure 1:**
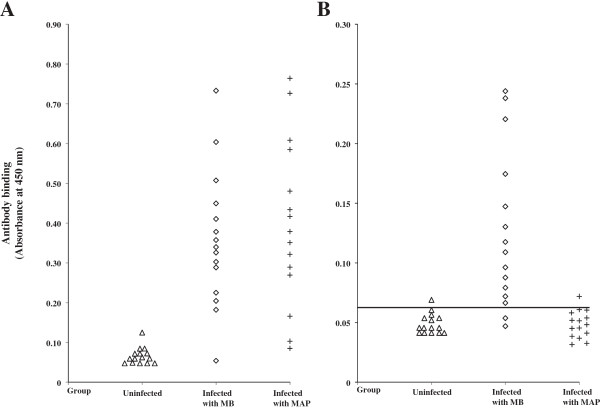
**Diagnostic performance of EVELISA.** Antibody binding was tested using 45 red deer serum samples, without **(A)** and with **(B)** absorption of cross reactive antibodies against MAP. The samples were divided in 3 groups (Uninfected (n = 15); Infected with *M. bovis* (n = 15); Infected with MAP (n = 15)). Each marker represents an average of duplicate measurements. To estimate tentative diagnostic sensitivity and specificity of the EVELISA test, cut-off value of 0.065 (horizontal line) was used **(B)**.

**Figure 2:**
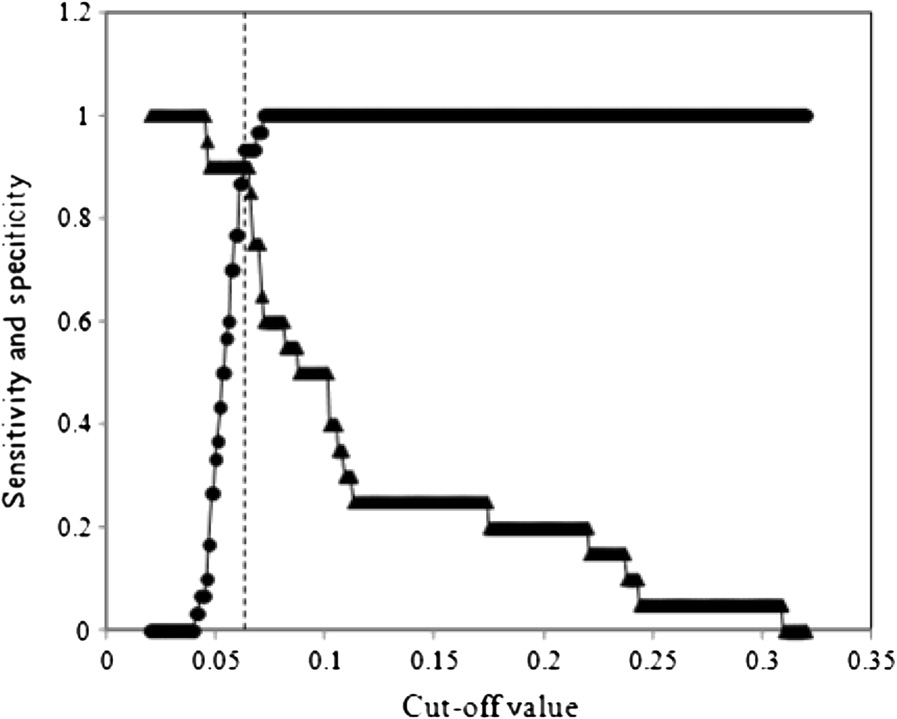
**Two-graph receiver operating characteristic analysis of the EVELISA data for determination of a cut-off value.** The data shown in Figure 2 were plotted on a graph having cut-off values on the X-axis and sensitivity (triangle)/specificity (circle) on the Y-axis. The dotted line indicates the selected cut-off value (0.065).

The performance of EVELISA test to detect anti-*M. bovis* antibodies in the sera of red deer was evaluated and compared with or without the use of MAP as an absorbent for cross-reactive antibodies (Figure 1A-B). Without pre-absorption of sera with MAP (Figure 1A), 14 out of 15 *M. bovis* positive samples (*M. bovis*-infected) showed higher antibody binding levels than those in any of uninfected samples (Uninfected). In the same figure, 13 out of 15 samples in the third group (MAP-infected) showed high antibody binding levels than those in the uninfected samples, showing a high rate of false positive reactions. In Figure 1B, antibody binding was tested after absorbing cross-reactive antibodies with heat-killed MAP bacilli. Antibody binding levels in the MAP-infected groups were significantly reduced. Two-graph receiver operating characteristic analysis was used to calculate a cut-off value of 0.065 (Figure 2). Anti- *M. bovis* antibodies were detected in 86.7% (13/15) of *M. bovis*-infected animals. Out of the 15 animals in the first group (Uninfected), 14 animals showed negative reaction with the EVELISA. The most significant difference was that antibody binding levels in the third group (n = 15, MAP infected) were reduced to the same levels as the first group (Uninfected) and only one sample in the MAP infected group showed antibody level higher than the cut-off value. We also tested 5 animals naturally infected with *M. bovis* and found that all the animals had higher antibody binding than the cut-off value (Mean ± standard deviation of absorbance at 450 nm = 0.127 ± 0.103).

## Discussion

Previous studies on developing ELISA for bTB in deer have used PPD (bovine and avian), MPB70 [4,25,38], and LAM [39,40]. Griffin *et al*. [35] have described the use of bovine and avian PPD in comparative ELISA format (B= > A) and suggested a sensitivity of 70% [35]. They have also reported an ancillary blood test (BTB) which is a composite test of both lymphocyte transformation assay and the bovine PPD ELISA. The BTB showed a high sensitivity of 94%; however, the test is a costly assay compared to ELISA and is used as an ancillary test to examine TST-reactors rather than for whole herd testing. LAM has also been used as an antigen in development of antibody based assays and to evaluate antibody response kinetics on experimental inoculation but it is cross reactive to antibody elicited by non-tuberculous mycobacteria [39]. Waters *et al*. [40] suggested the use of purified proteins to improve the specificity of antibody based assays [40].

Recently, other serologic tests have been developed – MAPIA, RT and dual-path-platform (DPP) VetTB assay – using *M. bovis*-specific antigens such as MPB83, ESAT6 and CFP-10 antigens [18,26,41]. Sensitivities of MAPIA and Cervid TB STAT-PAK were estimated to be 76.7% and 72.5%, respectively, by using sera of cervids (*C. elaphus*) experimentally infected with *M. bovis*[42]. Buddle *et al*. [28] compared the 2 lateral flow tests – CervidTB STAT-PAK and DPP VetTB assay and reported specificities of 83.8% and 91.4%, respectively [28]. Further, in a following study, higher sensitivities of CervidTB STAT-PAK (82%) and DPP VetTB (79%) were reported by Waters *et al*. [12] by using sera of *C. elaphus* naturally infected with *M. bovis*[12]. Boadella *et al*. [43] reported a sensitivity of 51% and specificity of 96% for fallow deer using bovine PPD ELISA. They also tested DPP VetTB assay and depending on the cut-off value selected, the sensitivity and specificity ranged from 62-71% and 88-95%, respectively [43]. Although these rapid tests show improved sensitivity and specificity, preparation of the highly purified recombinant proteins can be costly.

In this study, we used *M. bovis* antigens prepared simply by agitating the bacteria in an ethanol solution. Our previous studies showed that ethanol extract of MAP contains MAP-specific antigens and can be used to diagnose JD with high sensitivity [30,44]. We also showed that absorption of cross reactive antibodies in serum samples with *Mycobacterium phlei* improved specificity of the EVELISA test for JD [32]. This is similar to the observation in this study that specificity of EVELISA test could be improved by absorption of cross reactive antibodies by using environmental mycobacteria. After absorption of cross reactive antibodies with MAP, anti-*M. bovis* antibodies were detected in 86.7% of *M. bovis*-infected animals with only a minor false positive result in uninfected animals. Fourteen out of 15 samples from animals in the group (MAP infected) were tested negative by the EVELISA, indicating that a majority of antibodies reacting with MAP were removed by the absorption. Thus, the results of this study suggest that the EVELISA could diagnose bTB in MAP-infected red deer with minimum false positive results caused by antibodies reacting with MAP, encouraging further studies to validate the test using a larger number of samples obtained from red deer farms. In such studies, it would be important to test the samples with other *M. bovis* antigens (e.g. PPD, LAM and MPB70/83) to directly compare the performance of EVELISA with those of reported antigens.

## Conclusion

The findings presented above indicate that EVELISA could detect antibodies against *M. bovis*. However, characterization of immunodominant antigens in the ethanol extract and testing a larger number of samples for validation of the results are required. This study suggests that EVELISA can form a basis for development of a sensitive and specific test for bTB in deer.

## Abbreviations

bTB: Bovine tuberculosis; JD: Johne’s disease; M. bovis: *Mycobacterium bovis*; MAP: *Mycobacterium avium ssp. paratuberculosis*; ELISA: Enzyme linked immunosorbent assay; EVELISA: Ethanol Vortex enzyme linked immunosorbent assay; TST: Tuberculin skin test; PPD: Purified protein derivatives; LAM: Lipoarbinomannan; MAPIA: Multi-antigen print immune-assay; RT: Rapid test; DPP: Dual path platform.

## Competing interests

The authors declare that they have no competing interests.

## Authors’ contributions

SE conceived the study, carried out the statistics and designed the experiments. AW, REJ conducted the experiments. AW drafted the final manuscript with the help of SE. CM, JFTG, WRW, and JPB helped in designing the experiments and provided samples – reagents for the study. All authors read and approved the final manuscript.

## Authors’ information

AW was a graduate student (when the study was conducted) and SE an Associate Professor in the Center for Wildlife Health, Department of Forestry, Wildlife and Fisheries, University of Tennessee Institute of Agriculture, Knoxville, Tennessee. REJ is a Veterinary Student at the College of Veterinary Medicine, University of Tennessee, Knoxville. CGM and JFTG are senior researchers at AgResearch Invermay, New Zealand. Both, WRW is a Veterinary Medical Officer and JPB is a Research Microbiologist at USDA, Ames, IA.
